# An Exploratory Study: What Can Eye-Movements Tell Us About Phonological Awareness in Children with Intellectual Disabilities?

**DOI:** 10.3390/jemr19050115

**Published:** 2026-10-08

**Authors:** Ruth Sarimski, Peter Zentel

**Affiliations:** Education for Persons with Intellectual Disabilities and Inclusive Education, Ludwig Maximilian University of Munich, 80539 Munich, Germany; peter.zentel@edu.lmu.de

**Keywords:** phonological awareness, reading acquisition, children with intellectual disabilities, looking-while-listening paradigm, eye tracking

## Abstract

Word decoding as a foundational reading skill is significantly associated with phonological awareness in children with typical development as well as children with intellectual disabilities. Phonological awareness is often assessed using traditional behavioral methods, which may underestimate abilities, particularly in individuals who are difficult to test due to behavioral, communication, or attentional difficulties. This study tested whether an eye-tracking “looking-while-listening” paradigm provides an additional recording option which is appropriate and potentially informative to examine the cognitive process while solving phonological awareness tasks in the assessment of children with intellectual disabilities. Fifty-nine children with mild to moderate intellectual disabilities (aged 8–14 years) completed sound, syllable, and phoneme blending and segmentation tasks in paper-based and eye-tracking formats. Participants were classified as proficient or non-proficient based on behavioral performance in the paper-based format. Eye-tracking indices included proportional fixation duration, counts, revisits, and duration of the initial fixation (first fixation) on target, phonologically similar, and distractor images. Proficient participants showed a higher proportional total fixation duration and a higher frequency of fixation on target images, as well as greater attention to phonologically similar competitors than distractors, compared to non-proficient participants, who showed no such differentiation. The findings which we obtained in our exploratory study suggest that eye tracking is a feasible, process-oriented method for assessing phonological awareness in children with intellectual disabilities and may capture cognitive mechanisms not reflected in traditional assessment procedures. This assumption should be pursued further through carefully designed experimental studies.

## 1. Introduction

Comprehensive research has demonstrated that students with intellectual disabilities encounter more significant challenges in developing word decoding than their typically developing peers (e.g., [1]). However, there is considerable variability in the reading abilities among students with intellectual disabilities.

The current literature on students with intellectual disabilities suggests that decoding difficulties are associated with deficits in phonological awareness [2,3,4,5], letter–sound knowledge [4,6], and rapid automatized naming [2,5,7,8,9]. The predictors of decoding abilities in students with intellectual disabilities closely resemble those identified in earlier research on younger, typically developing children.

This exploratory study focuses on phonological awareness. Phonological awareness is an umbrella term encompassing the ability to manipulate individual sounds within words, such as phoneme segmentation and blending, as well as broader awareness of syllables, rhymes, and onsets. Previous research on reading disabilities has identified core deficits in phonological processing as a primary source of difficulty in decoding and spelling written words [10]. Phonological awareness is also an area of marked difficulty in children with Down syndrome, Williams syndrome, or Fragile X syndrome compared to typically developing children matched on mental age, verbal ability, language skills, and reading level [11,12,13,14,15].

However, most phonological awareness tasks are scored based on accuracy and thus capture only whether children respond correctly. Performance on such tasks depends on an individual’s ability to produce prompt and reliable overt responses to task instructions (e.g., pointing to an image or answering questions). Consequently, these measures may confound phonological awareness with difficulties in communication, social reciprocity, attention, or motor functioning, potentially underestimating abilities in hard-to-test populations, such as children with low willingness to cooperate [16]. Additionally, accuracy-based scores (correct versus incorrect) provide no information about how effortful or challenging task performance is for the individual.

Eye-tracking methods provide a novel means of objectively capturing cognitive processing without requiring overt behavioral responses, particularly in difficult-to-test populations. Eye-tracking measures can index how children allocate attention across different sources of input, as well as their referential decisions, that is, where they direct their gaze to what they consider meaningful or to what they believe is being referred to [16].

Eye-tracking methods have been widely used to assess language comprehension in young children with typical development as well as in clinical populations. The looking-while-listening paradigm [17] provides a controlled experimental approach for assessing real-time language processing. In a standard task, multiple images are presented on a screen while an auditory prompt is played (e.g., “Where is …?”). In contrast to traditional behavioral assessments, which typically rely on dichotomous pass/fail scoring, eye-gaze measures provide continuous, graded information about the accuracy and efficiency of language processing. Increased fixation on the named target image over the course of a trial is taken as an index of comprehension and can be used to estimate vocabulary knowledge.

For example, the looking-while-listening paradigm has been applied in studies of children with language impairment [18] and young children with autism spectrum disorder (e.g., [19]). Moreover, eye-gaze patterns during receptive language processing have been shown to correlate with traditional measures of language ability in children with Autistic Spectrum Disorder (ASD), supporting the validity of eye-gaze measures for the assessment of receptive language [20,21].

Eye tracking has become an established method for examining early reading acquisition in both typically developing children and children with dyslexia. Key eye-movement parameters include first fixation duration, total fixation duration, fixation count, and regressions, all of which provide information about the allocation of visual attention during reading [22,23]. First fixation duration reflects the initial stages of word processing, including early decoding and lexical access, whereas total fixation duration captures the cumulative time spent processing a word and is assumed to index both lexical and higher-level integrative processes. Regressions, defined as backward eye movements to previously inspected text, are commonly interpreted as markers of processing difficulties or efforts to resolve uncertainty. Overall, longer proportional total fixation durations and increased fixation counts are indicative of greater cognitive and attentional demands during visual, orthographic, and linguistic processing.

Age-related changes in eye-movement characteristics have been shown to correlate with reading outcomes [24]. Recent research has examined how eye-movement patterns evolve during reading development. In the earliest stages of reading acquisition, decoding is typically characterized by slow and effortful processing based on small orthographic units, such as letter-by-letter reading. This stage is associated with longer fixation durations and a higher frequency of fixations, including both initial fixations and re-fixations. As reading proficiency increases, word recognition becomes progressively automatized, resulting in shorter fixation durations and fewer fixations overall [25,26,27].

Eye-movement characteristics also differ between children with age-appropriate reading skills and those with dyslexia. In a review of eye-tracking studies on reading in children with dyslexia, Premeti et al. [28] reported that individuals with reading difficulties exhibit longer fixation durations, a greater number of fixations, a lower proportion of skipped words, and shorter saccades than typically developing readers. Furthermore, Pereira et al. [29] demonstrated that eye-tracking measures, when combined with neuropsychological indicators, improved the prediction of reading abilities in children with dyslexia and attention-deficit/hyperactivity disorder (ADHD). Beyond dyslexia, eye tracking has also been applied to the study of reading in other neurodevelopmental populations. For example, Andreou and Raxioni [30] reviewed eye-tracking studies investigating reading processes in children and adults with autism spectrum disorder, highlighting the utility of eye-movement measures for understanding individual differences in reading-related cognitive processing.

Although eye tracking has been widely used to investigate reading development in typically developing children and in populations with reading difficulties, its application to children with intellectual disabilities remains largely unexplored. To the best of our knowledge, no previous study has employed eye-tracking methods to examine reading acquisition and associated difficulties in this population [23]. Specifically, no study has yet examined the use of eye-tracking technology for assessing phonological awareness in children with intellectual disabilities. The present exploratory study aims to address this gap by investigating the underlying cognitive processing mechanisms that children with intellectual disabilities employ when completing phonological awareness tasks. Specifically, we proceed from the assumption that children who possess phonological awareness differ from children who do not in the distribution of their visual attention in a looking-while-listening paradigm and that these children will consider images which are phonologically similar to the target longer compared to images which have no similarity with the target indicating greater processing effort to discriminate between target and a phonologically similar alternative.

In order to explore this assumption, we propose the following hypotheses:

1. In a looking-while-listening paradigm, students who respond correctly on phonological awareness tasks will show higher proportional total fixation duration on the target image compared to the mean proportional fixation duration across non-target images;
2. In a looking-while-listening paradigm, students who respond correctly on phonological awareness tasks will show a higher frequency of fixations on the target image compared to the mean proportional fixation duration across non-target images;
3. In sound blending, syllable blending, and phoneme segmentation tasks, students who respond correctly on phonological awareness tasks will exhibit higher proportional total fixation duration, higher fixation counts, and more revisits to phonologically similar competitor images compared to distractor images.

## 2. Materials and Methods

### 2.1. Participants

Sixty-nine children with mild to moderate intellectual disabilities aged 8–14 years participated in the study. Participants were recruited from special schools in southern Bavaria, Germany. Recruitment and assessment were conducted across six sites. As part of the screening procedure, the primary researcher conducted interviews with teachers to assess the specific conditions and suitability of each participant for inclusion. The final sample comprised children without vision or hearing impairments and without motor limitations that could significantly hinder participation in the testing sessions (e.g., severe visual impairment, severe hearing loss, or inability to sit upright without assistance and maintain head control).

Children with severe autistic behaviors were excluded, as it was anticipated that they would be unable to participate reliably due to behavioral difficulties. All exclusion criteria were based on teacher reports. Where available, information regarding medical diagnoses was also provided by teachers. The study was conducted in accordance with ethical approval granted by the LMU Ethics Committee. Informed consent was obtained from all participants involved in the study by their legal guardians.

Of the children initially enrolled, ten were excluded during the assessment process. Exclusion criteria comprised severe cooperation difficulties (two children), technical failures of the eye-tracking system (two children), and prolonged illness occurring after the first assessment session (one child). Five children were excluded because they were unable to successfully complete the eye-tracking procedure (see below).

The final group consisted of 59 children (29 boys and 30 girls). The mean age was 134.15 months (SD = 18.12). Mean non-verbal IQ was 57.81 (SD = 10.44), and mean developmental age on a word comprehension test was 55.01 months (SD = 11.59). Table 1 presents descriptive statistics for all relevant sample characteristics.

### 2.2. Materials and Procedure

Testing took place in a small, quiet room and comprised three assessments of nonverbal intelligence, word comprehension, and phonological awareness. Nonverbal intelligence and word comprehension were assessed first using standardized test instruments. After completion of the testing, phonological awareness was assessed in two different formats. First, 12 tasks assessing phonological awareness (see below) were presented on a screen, and the participants’ eye movements were recorded. Then, the same tasks (with a different arrangement of the images) were presented in a paper-based version. Participants were asked to point to the correct image.

*Nonverbal intelligence*: Nonverbal reasoning was assessed using the Raven’s Coloured Progressive Matrices 2 (RCPM; [31]). The RCPM consists of three sets of 12 matrices of increasing difficulty. Participants are required to identify the missing element in a visual pattern, typically presented in matrix format, by selecting the correct response from a set of alternatives. Responses are given by pointing to the chosen image. The RCPM is suitable for children from 4 years of age and for individuals with intellectual disabilities. It demonstrates good test–retest reliability and high internal consistency. A German adaptation is available. Standard scores were derived based on chronological age using normative data collected in Germany and other European countries.

*Word comprehension:* Receptive vocabulary was assessed using the Peabody Picture Vocabulary Test—Fourth Edition (PPVT-4; [32]). This test requires children to select the picture that corresponds to a spoken word (e.g., “Point to ‘ball’”). The PPVT-4 comprises 228 items and is administered until a predefined termination criterion is reached. A German version with representative norms is available.

*Phonological Awareness:* Phonological awareness was assessed using a looking-while-listening paradigm. The tasks were designed based on examples provided by Light and McNaughton [33] and were administered twice. To examine sound blending, syllable blending, and phoneme segmentation skills in this format, four two-syllable words and selected corresponding pictures were compiled into 12 items. Each presentation stimulus consisted of four pictures arranged in a grid format, with one target image and three non-target images. The images were presented on a white background divided into four equal quadrants. All pictures were matched for size and centered within their respective quadrants (10 × 10 cm), with a 10 cm horizontal and 5 cm vertical gap between images. The location of the target image was randomized across trials. For each stimulus set, visual presentation was paired with a standardized auditory prompt naming the target (i.e., sounds, syllables, or phonemes).

Stimuli were presented on a computer screen connected to a stationary remote Tobii Pro X3-120 eye tracker (Tobii Pro, Danderyd, Sweden), which employs a video-based corneal reflection technique to record participants’ eye movements during task performance. The participants’ heads were not fixed; that means, the participants were allowed to move their heads during the task. When viewing the stimuli on a 27-inch screen, the participants sat on a chair at a distance of 60 cm from the screen (screen resolution 2560 × 1440 pixel). The eye-tracker recorded the X- and Y-coordinates of the eye position and pupil diameter. The eye-tracker measurement accuracy was within an average deviation of 0.4–0.5° of visual angle between the actual and recorded gaze positions. The sampling rate for eye tracking is 120 Hz.

All eye-tracking data were recorded and analyzed using iMotions software (version 9.3.8; 2023). Default software settings were applied throughout data collection and analysis. The iMotions software recorded the fixation data to a pre-set area of interest (AOI). An AOI was manually outlined for each image. Using the four images as AOIs we analyzed gaze behavior within each of the four quadrants. The first dependent measure was the proportion of total fixation duration directed to the target AOI (i.e., total fixation duration on the target divided by the total stimulus presentation time, i.e., 12 s). We then compared the proportional fixation duration on the target image with the proportional fixation duration on the phonological competitor (i.e., the image representing a word phonologically similar to the target) and with the proportional fixation duration on the distractor images (averaged across the two distractors).

In addition to fixation duration, further indicators of the distribution of students’ attention were extracted and analyzed: fixation count (i.e., the total number of fixations within each AOI per trial), revisit count (i.e., the number of times a participant returned their gaze to a given AOI), and first fixation duration (i.e., the duration of the initial fixation within an AOI).

Prior to the administration of the phonological awareness tasks, each participant completed a nine-point calibration procedure. The standard calibration protocol provided by iMotions was used, requiring participants to visually track a red circle that sequentially appeared at nine locations on the screen. Calibration quality was automatically evaluated by the software and classified as “excellent,” “good,” or “poor.” If calibration quality was rated as poor, the procedure was repeated for up to three attempts. Final calibration quality was classified as excellent for 13.6% of participants, good for 47.5%, and poor for 39.0%.

Despite suboptimal calibration in some participants, all included datasets met predefined criteria for data completeness and gaze-signal availability during task performance. These participants were therefore retained in the analyses. This decision was based on the assumption that fluctuating attention and increased irritability are common among students with intellectual disabilities and that suboptimal calibration would not necessarily invalidate the data obtained during subsequent task presentation. Consequently, no participant was excluded solely on the basis of poor calibration quality. However, when the software was unable to determine calibration quality altogether (e.g., due to persistent gaze aversion), the assessment session was terminated and the participant was excluded from the study. Five children were excluded for this reason. 

For the final group, the experimental trials to assess phonological awareness were initiated via researcher-controlled button press. Participants were instructed to listen carefully and “catch the target image with your eyes”. Presentation time (12 s) and verbal instructions were standardized across participants. The choice of presentation time was based on a pilot study conducted to assess the feasibility of the procedure for measuring eye movements with six typically developing children (7–8 years old). These participants were not included in the final sample. The presentation duration for our main study was set at twice the average time these children required to decide on the target image.

In a second trial, the same stimuli were presented in a paper-based format, and the student was asked to point to the target image. The position of the target images was randomly changed compared to the first trial. The investigator presented the next item as soon as the child had indicated their choice by pointing to one of the images.

In the sound blending task, the researcher presented a sequence of phonemes that together formed a spoken word. The student was asked to blend these sounds to identify the target word and to indicate which of four images represented it. One image corresponded to the target word, a second depicted a phonological competitor (i.e., a rhyming word with a different initial phoneme) and the remaining two served as unrelated distractors.

To assess syllable blending, the same procedure was repeated using syllables instead of individual phonemes. Again, one image corresponded to the target word, a second depicted a phonological competitor (i.e., a word with the same initial phoneme and the same syllable structure) and the remaining two served as unrelated distractors.

Phoneme segmentation was assessed by having the administrator produce a target sound (rather than a letter name). The student was then asked to identify which image represented a word beginning with that sound by examining each image, segmenting the initial sounds of the corresponding words, and selecting the appropriate target. As in the previous tasks, one image represented the target, one a phonological competitor (i.e., a rhyming word with a different initial phoneme), and the remaining two served as unrelated distractors.

Scores from the sound blending, syllable blending, and phoneme segmentation tasks were combined into a composite measure of phonological awareness (summary score “phonological awareness”).

Examples of the tasks are provided below:

For *sound blending*, the phoneme sequence *S–o–n–n–e* (English: *sun*) was presented, alongside four images: *Sonne* (sun; target), *Tonne* (bin; phonological competitor), *Sahne* (cream), and *Seife* (soap). In another example, the sequence *M–a–m–a* (English: *mummy*) was presented with the images *Mama* (mummy; target), *Lama* (llama; phonological competitor), *Mine* (mine), and *Möhre* (carrot).

For *syllable blending*, the sequence *Af*–fe (English: *monkey*) was presented with the images *Affe* (monkey; target), *alle* (all; phonological competitor), *Ente* (duck), and *Erbse* (pea). In another example, *Bri–lle* (English: *glasses*) was presented alongside *Brille* (glasses; target), *Berge* (mountains; phonological competitor), *Straße* (street), and *Flasche* (bottle).

For *phoneme segmentation*, the target sound */t/* was presented, indicating that the word begins with this phoneme. The corresponding images were *T/opf* (English: pot; target), *K/opf* (head; phonological competitor), *F/isch* (fish), *S/aft* (juice). In another example, the phoneme */l/* was presented. The corresponding images were *l/aufen* (English: to run; target), *k/aufen* (to buy; phonological competitor), *f/ahren* (to drive), *r/eiten* (ride).

### 2.3. Data Analysis

Descriptive statistics were computed for all variables. We defined “proficient performer” as students who correctly solved at least eleven out of twelve tasks, indicating adequate phonological awareness, and “non-proficient performer” as students who solved fewer than eleven tasks correctly. This classification was based on the scores obtained from the paper-based version of the phonological awareness test.

In a second step, we analyzed proportional total fixation duration, fixation counts, revisit counts, and first fixation duration on target stimuli, phonologically similar images, and distractor images for both subgroups (“proficient performer” and “non-proficient performer”). These measures were derived from the eye-tracking version of the tasks. Group differences were assessed using Student’s *t*-test.

As a third step, we conducted a repeated-measures analysis of variance (ANOVA) to examine differences between target stimuli, images representing phonologically similar words, and unrelated distractors. The Greenhouse–Geisser correction was applied to account for violations of the sphericity assumption. The assumptions of homogeneity of error variances (Levene’s test) and covariances (Box-M-test) required for a mixed-design ANOVA were not satisfied. Therefore, in accordance with Cohen’s [34] recommendation, the mixed design was broken down, and separate one-way repeated-measures analyses of variance were conducted for each of the study groups instead to interpret the differences between the target, the phonologically similar image, and the distractor within each group [35].

## 3. Results

### 3.1. Accuracy Rates in Phonological Awareness Assessment

Table 2 presents the accuracy rates for the three task groups as well as the composite score for phonological awareness (summary score “phonological awareness”). On average, children correctly solved 7.28 out of 12 tasks in the paper-based version. Seventeen children were classified as “proficient performer”, i.e., they correctly solved at least 11 of the 12 tasks. Forty-two children were classified as “non-proficient performer”. The distribution of solution frequencies in the group of “non-proficient performers” was very heterogeneous. Five children solved a maximum of six items correctly. The other participants in the group of “non-proficient performers” solved seven to ten items correctly.

Performance on the phonological awareness tasks was significantly correlated with children’s age (*r* = 0.32, *p* = 0.012), raw scores on the non-verbal intelligence test (*r* = 0.46, *p* < 0.001), and developmental age in the receptive vocabulary test (*r* = 0.35, *p* = 0.007).

### 3.2. Eye-Movement Measures (Comparison of Proficient and Non-Proficient Participants)

Table 3 presents the distribution of revisits, fixation counts, proportional total fixation duration, and first fixation duration for correct images, phonologically similar items, and distractors.

The comparison between “proficient” and “non-proficient” participants revealed significant differences in fixation counts (*t* (57) = −3.58, *p* < 0.001) and proportional total fixation duration (*t* (57) = −3.28, *p* < 0.001). Target images were fixated longer and more frequently by “proficient” participants. First fixation duration was longer on phonologically similar competitors (t (57) = 1.69, *p* < 0.05). No significant differences were observed for the number of revisits. Accordingly, Hypothesis 1 and Hypothesis 2 can be considered supported. The proficient performer group allocated their attention more frequently to the correct target images and their proportional total fixation duration was longer. Notably, this pattern was also observed—to a significantly lesser extent—in the non-proficient performer group. This finding is attributable to the group’s substantial heterogeneity in solution frequency. Most children classified within this group solved some items correctly; however, they could not be reliably characterized as possessing phonological awareness.

**Figure 1:**
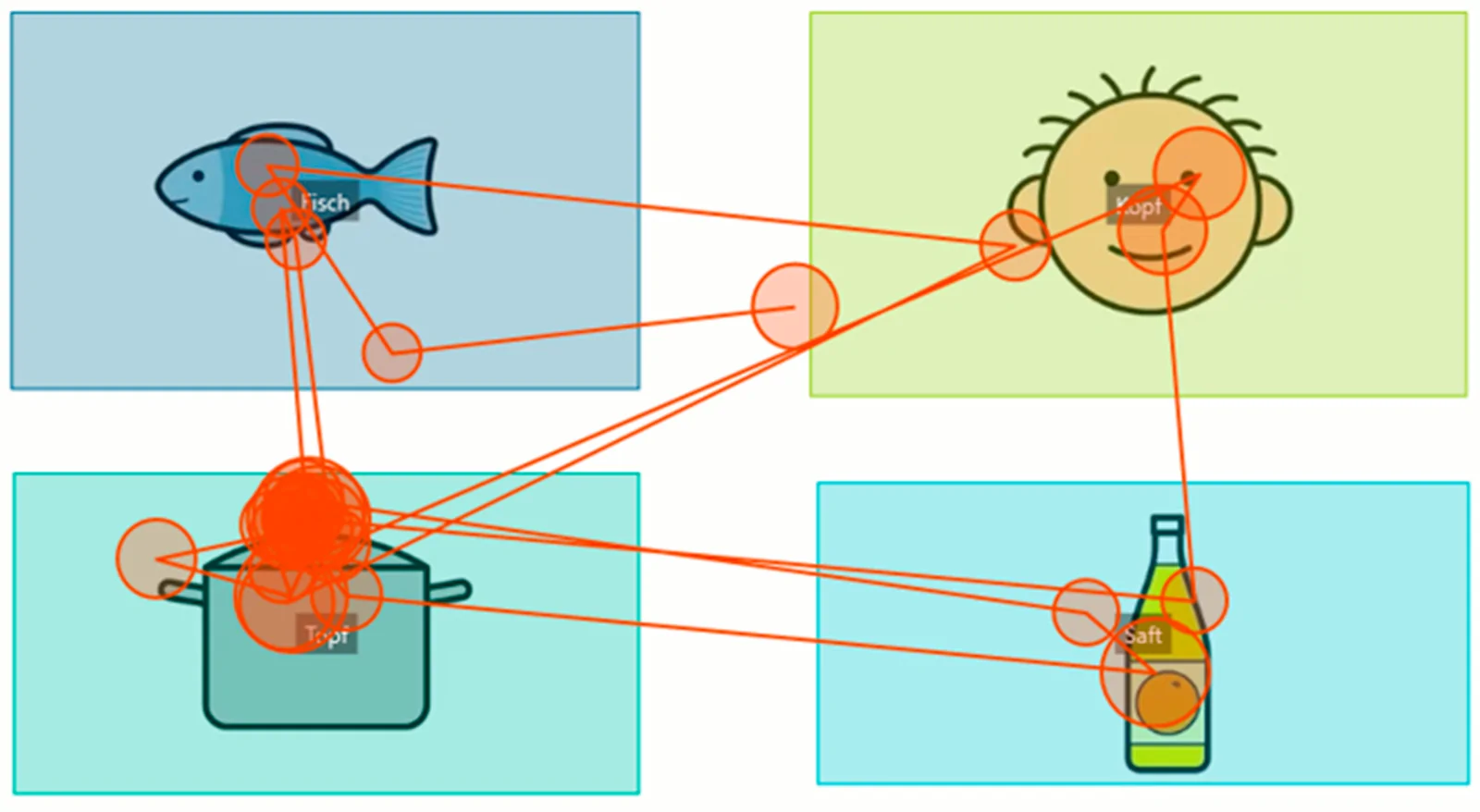
Example proficient performer: Gaze plot; phoneme segmentation (target word: Topf, engl.: pot; phonologically similar: Kopf (head), visualization of the temporal sequence of eye movements while viewing the stimulus; circle size reflects longer fixation duration.

**Figure 2:**
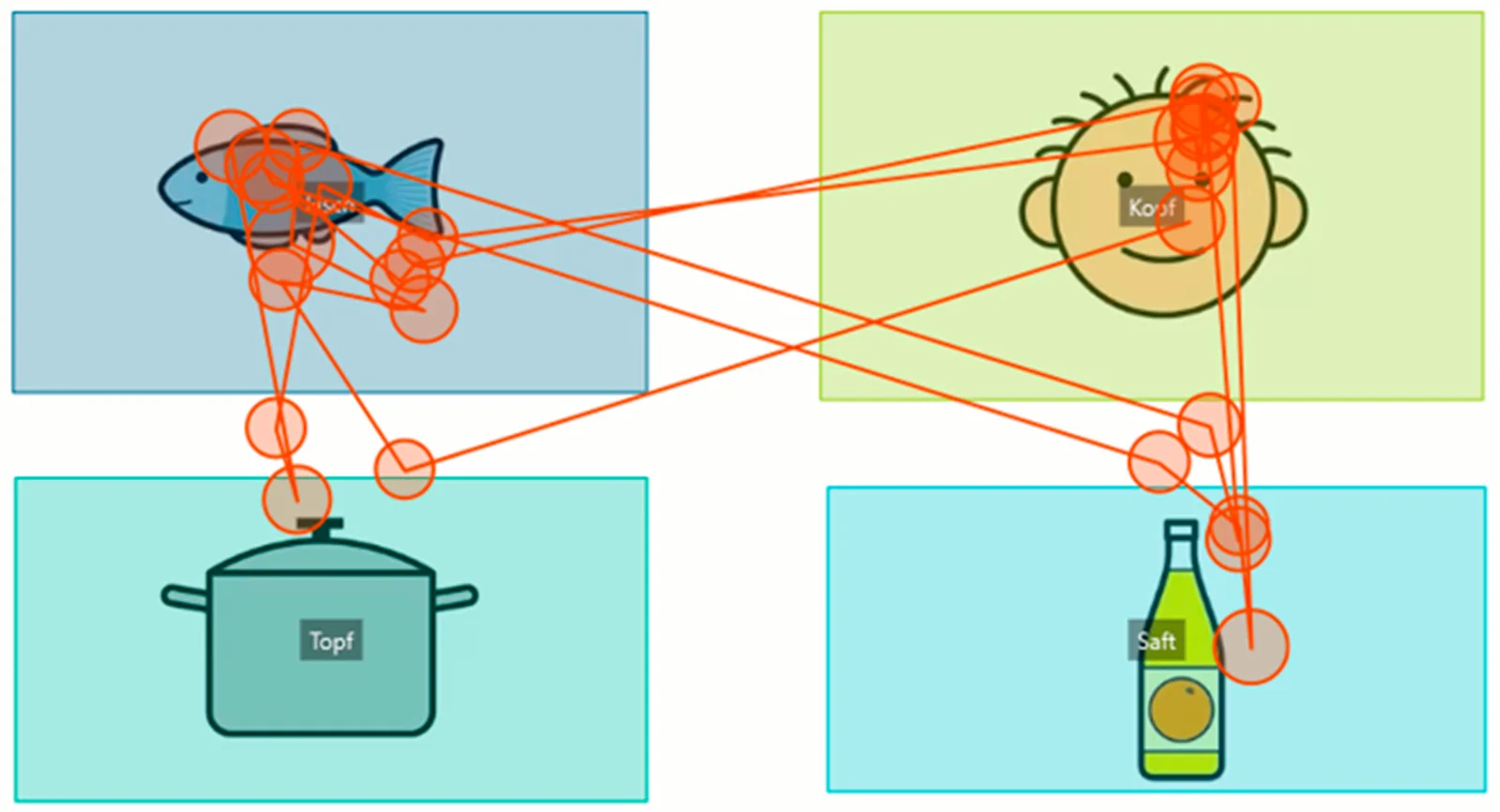
Example non proficient performer: Gaze plot; phoneme segmentation (target word: Topf, engl.: pot; phonologically similar: Kopf (head) visualization of the temporal sequence of eye movements while viewing the stimulus; circle size reflects longer fixation duration.

One example to illustrate gaze performance patterns in both groups are listed below (see Figure 1 and Figure 2).

Separate repeated-measures analyses of variance (ANOVAs) were conducted for the groups of proficient and non-proficient participants. Within the proficient group, a significant effect was observed for count measures, *F* (1.05, 16.94) = 41.73, *p* < 0.001, indicating a significantly higher number of counts for the target images, whereas no difference was found between phonological competitors and distractors (*p* = 0.15). Regarding proportional total fixation duration, the ANOVA likewise revealed a significant effect, *F* (1.05, 16.72) = 30.35, *p* < 0.001. Pairwise comparisons further indicated a significant difference between phonologically similar competitors and distractors, *p* < 0.046. Regarding first fixation duration, the ANOVA revealed no significant effect (F (1.50, 24.00) = 1.33, *p* = 0.27).

Within the non-proficient group, the analysis of variance for count measures also revealed a significant effect, F (1.21, 51.37) = 87.93, *p* < 0.001. Pairwise comparisons indicated that phonologically similar competitors differed from distractors, with phonologically similar competitors being fixated more frequently, *p* = 0.013. With regard to proportional total fixation duration, the ANOVA likewise showed a significant effect in this group, *F* (1.14, 47.05) = 55.51, *p* < 0.001. However, in contrast to the proficient group, phonologically similar competitors and distractors did not differ significantly, *p* = 0.076. Regarding first fixation duration, the ANOVA revealed a significant effect (F (1.69, 66.63) = 3.57, *p* = 0.04), but no difference between phonologically similar competitors and distractors (*p* = 0.82).

Hypothesis 3 can therefore be considered partially supported. Children with reliable phonological awareness skills (“proficient” participants) not only directed their attention longer toward the target image, but also showed a difference in fixation duration between phonologically similar competitors and distractors. Specifically, similar competitors received a greater proportion of total fixation duration than distractors.

## 4. Discussion

In this exploratory study, phonological awareness as a foundational skill for reading acquisition was examined in 59 children with intellectual disabilities. The tasks included items assessing sound blending, syllable blending, and phoneme segmentation. These tasks were first administered on a screen connected to an eye-tracking device and subsequently presented in a paper-based format.

The analyses showed that children classified as “proficient performers”, i.e., children who solved eleven or 12 items in the paper version correctly, also had fixated the target significantly longer and more frequently in the first assessment phase presenting the items on the screen—in comparison to children who had not (yet) developed these phonological skills reliably. Moreover, these children directed their attention longer toward images whose labels were phonologically similar to the target word than toward distractor images. Such a difference was not observed in children who were less successful in solving the phonological awareness tasks.

First, the findings of this exploratory study suggest that a looking-while-listening paradigm can be successfully applied with children with intellectual disabilities—in this case, to assess phonological awareness—in order to differentiate between proficient and non-proficient participants. Our results can be placed in the context of findings from studies involving children with typical development which indicate that eye tracking can be used as a tool to test phonological awareness skills (e.g., [36,37]).

Second, this type of task design may provide insight into which stimuli were more difficult for the children to discriminate, thereby offering a potentially informative approach to investigate the underlying cognitive processing involved in solving tasks designed to assess phonological awareness. When presented with four images, those whose labels were phonologically similar to the target word were fixated longer than distractor images. This pattern may indicate that these images were strongly considered as potential correct responses so that children engage in phonological analysis by isolating individual sounds and comparing them to those of the target word. Children who had not yet developed reliable phonological awareness skills may not appear to perceive such phonological similarity and were, therefore, not (yet) able to engage in the same internal processes of phonological analysis, working memory processing, and comparison with the target word.

This result can be interpreted in light of previous findings indicating that the frequency and duration of fixations on stimuli in eye-tracking tasks reflect the level of difficulty associated with task performance. In particular, fixation duration serves as an index of the amount and quality of cognitive effort allocated to the processing and evaluation of stimuli [22,38,39].

To date, only a limited number of studies have examined eye movements in children with developmental disorders using a looking-while-listening paradigm. Bavin et al. [19] employed this paradigm to assess the efficiency of lexical processing in children with symptoms of autism spectrum disorder. In their study, one image depicted an object matching the meaning of the word, one showed an object whose label was phonologically similar to the target word, and two additional images served as unrelated distractors. Thus, the images varied in the level of lexical processing demands required for successful discrimination. Baron et al. [40] likewise employed a looking-while-listening paradigm to investigate the effects of varying levels of lexical processing demands on word discrimination. Specifically, they examined whether consistency between spelling and pronunciation (“orthographic consistency”) facilitated children’s ability to identify, among four images, the one whose label ended with the same phoneme as a target word. This phonological segmentation task, as a subcomponent of phonological awareness, was administered to children with and without reading difficulties. Again, this study demonstrated that both fixation frequency and duration can serve as indicators of task difficulty in visual discrimination tasks.

Within this framework, the findings of the present study can be interpreted. They suggest that phonological similarity leads children who already possess phonological analysis skills to engage in more prolonged processing of a stimulus in order to discriminate it from the target word, compared to distractor images that do not share phonological features with the target.

## 5. Limitations

Nevertheless, the interpretation of these findings remains tentative. Several limitations must be considered. A first limitation concerns the selection of images used in the tasks. One potential factor influencing fixation duration, fixation frequency, and the number of revisits is the visual salience of the distractor images. For instance, in a sound blending task requiring discrimination among “Mond” (eng.: moon)—“Bein” (engl.: leg)—“Maul” (engl.: mouth)—“mein” (engl.: mine; with “mein” as the target), a widely opened mouth was used as a distractor. It cannot be ruled out that such stimuli attracted children’s visual attention, thereby affecting their allocation of gaze to the target stimulus. Future studies should therefore control for the visual salience of the presented images in advance to ensure that observed effects are not confounded by differences in perceptual attractiveness. Another limitation has to be acknowledged. The choice of the images in the tasks was not standardized for complexity, familiarity, naming consistency, and word frequency. Each of these characteristics of the stimuli may have some influence on the allocation of visual attention and should be considered in future studies.

The fixed order of administration (screen-based assessment preceding the paper-based version) represents a potential limitation of the study. Nevertheless, it seems unlikely that participants’ performance on the paper-based task was markedly enhanced by prior exposure to the stimuli, as the images were presented in a different arrangement across the two formats. Thus, any practice effects are expected to have been minimal.

One specific limitation of the eye-tracking procedure should be acknowledged. We standardized the presentation duration of the stimuli to 12 s per item. This decision was informed by a pilot study with typically developing first-grade children, in which a presentation time of approximately 6–8 s appeared sufficient for making a choice. An alternative approach would have been to require children to indicate their response explicitly or to allow them to proceed by pressing a button. However, both options would have involved motor responses that might have diverted attention away from the visual stimuli. Given the relatively limited attentional capacities of children with intellectual disabilities, this was considered likely to compromise the reliability of the eye-tracking measurements. It cannot be ruled out that some children might have correctly identified the target stimulus if more than 12 s had been available to decode the spoken word and process the visual display.

A further limitation concerns the grouping strategy used in the present study. The distinction between “proficient performers” and “non-proficient performers” was based on a pragmatic criterion rather than on established theoretical or empirical benchmarks. Specifically, phonological awareness was assumed to be present when participants answered at least 11 of the 12 items correctly. Although this cutoff appeared reasonable for the purposes of the current analysis, it should be regarded as tentative.

Future research should aim to recruit larger samples that allow the formation of more homogeneous groups. In particular, it would be informative to compare the eye-movement patterns of children who perform at ceiling level with those of children who are unable to solve any, or only very few, of the phonological awareness tasks. Such a design would provide a more robust basis for examining associations between eye-movement characteristics and phonological awareness.

Finally, it must be acknowledged that, while analyses of fixation duration, fixation frequency, and the number of revisits allow us to infer the mental effort participants exerted to complete each task, these measures do not provide direct evidence of the specific internal cognitive processes involved. We can assume that participants engaged in internal processing that can be described as “say it in your head” (subvocal rehearsal). The present findings suggest this assumption but our interpretation should remain tentative, because the tasks we used combine phonological awareness, spoken vocabulary comprehension and visual search ability. That means, that it cannot be determined from our design direct and unequivocal whether, in these phonological awareness tasks, the differences in proportional total fixation duration reflected subvocal rehearsal of the image labels, their comparison, and selection of the correct target image in the process of solving phonological awareness tasks. It cannot be ruled out that individual differences in vocabulary comprehension or executive control had some influence on the results.

With regard to the use of eye tracking in analyzing cognitive processes in children with intellectual disabilities, it should be emphasized that for the majority of participants, eye-tracking sessions could be conducted successfully. Most children were able to be positioned effectively in front of the eye-tracking screen, and dropout due to insufficient cooperation with the device was low. Nevertheless, it should be noted that eye-tracking procedures are more susceptible to technical disruptions than paper-and-pencil assessments, which may have affected overall data quality. In several sessions, repeated software failures required the task to be restarted, temporarily influencing children’s attention, motivation, and cooperation.

In addition, calibration and re-calibration procedures proved challenging for some participants, likely due to increased variability in attention span and motor activity, which is typical in this population. Although calibration was repeated, when necessary (up to three consecutive attempts), the evaluation software rated calibration quality as “poor” in 39% of the sample. Even though we have justified the inclusion of these participants as well, this must be regarded as a potential limitation on data quality. An increased rate of calibration difficulties has also been reported in eye-tracking studies involving children with severe neurodevelopmental disabilities [41]. Potential contributing factors include difficulties with sustained fixation, less precise smooth-pursuit movements, and involuntary saccades, which may be more common in some children with neurodevelopmental disabilities. Because conventional calibration algorithms require participants to fixate accurately on a target for a certain period of time, unstable or variable gaze patterns may result in calibration failure or reduced calibration accuracy. In addition, standard calibration procedures typically require participants to visually follow targets as they move across the screen. For some children with intellectual disabilities, these procedures may provide insufficient motivation to consistently attend to and follow the calibration targets as instructed. Future studies could therefore consider using more engaging stimuli instead of standardized calibration targets, such as preferred cartoon characters or animal images, potentially accompanied by auditory cues, that participants are required to visually follow during calibration.

Despite these limitations, the study demonstrates that eye-tracking methodology is a valuable tool for analyzing prerequisite skills for reading acquisition in children with intellectual disabilities. Eye tracking is a process-oriented method for assessing phonological awareness in children with intellectual disabilities and may capture cognitive mechanisms not reflected in traditional assessment procedures. Although the present findings are promising, further research is required to evaluate the validity and reliability of this assessment approach before it can be recommended as a supplementary method for examining reading-related skills and challenges in children with intellectual disabilities.

## Figures and Tables

**Table 1:** Sample characteristics.

	*n*	%
Gender		
Boys	29	49.2
Girls	30	50.8
Age group		
8–10	14	23.7
11–12	25	42.4
13–14	20	33.9
Years of schooling		
2–3	19	32.2
4–5	19	32.2
6–8	21	35.6
Diagnoses		
Unknown etiology	20	33.9
Autism spectrum disorder	12	20.3
Down syndrome	10	16.9
Other syndrome	9	15.3
Cerebral palsy (CP)	6	10.2
Other	2	3.4

**Table 2:** Accuracy rates in phonological awareness tasks.

	Min–Max	Mean	Standard Deviation
Sound blending	0–7	3.93	1.21
Syllable blending	0–3	2.38	0.69
Sound Segmentation	0–2	0.96	0.76
Phonological awareness	0–12	7.28	1.87

**Table 3:** Eye-movement measures in phonological awareness tasks.

	Proficient Performer (17)		Non-Proficient Performer (42)		*p*
	Mean	SD	Mean	SD	
Revisit target	7.38	2.96	6.82	3.76	0.29
Revisit similar	4.83	3.05	4.50	2.82	0.34
Revisit distractor	4.42	2.53	3.75	2.11	0.15
Fixation count target	44.28	17.14	30.25	11.93	<0.001
Fixation count similar	14.96	6.09	14.90	6.15	0.48
Fixation count distractor	13.58	5.76	13.14	4.50	0.37
Proportional total fixation duration—target (%)	30.79	16.59	18.63	11.13	<0.001
Proportional total fixation duration—similar (%)	8.37	4.63	7.64	4.09	0.27
Proportional total fixation duration—distractor (%)	6.78	3.41	6.79	3.15	0.49
First fixation duration—target (ms)	573.26	310.85	513.91	212.28	0.20
First fixation duration—similar (ms)	534.43	246.61	445.95	148.11	0.047
First fixation duration—distractor (ms)	471.54	160.69	450.72	187.09	0.34

## Data Availability

The data presented in this study are openly available in OSF at https://doi.org/10.17605/OSF.IO/Q3EKA.
